# Amyloid β Oligomeric Species Present in the Lag Phase of Amyloid Formation

**DOI:** 10.1371/journal.pone.0127865

**Published:** 2015-05-29

**Authors:** Martin Wolff, Dmitry Unuchek, Bo Zhang, Valentin Gordeliy, Dieter Willbold, Luitgard Nagel-Steger

**Affiliations:** 1 Institut für Physikalische Biologie, Heinrich-Heine-University Düsseldorf, Düsseldorf, Germany; 2 Institute of Complex Systems, Structural Biochemistry (ICS-6), Research Centre Jülich, Jülich, Germany; 3 Dep. of Molecular and Chemical Physics, Moscow Institute of Physics and Technology, Dolgoprudniy, Russian Federation; 4 Institute of Complex Systems, Neutron Scattering (ICS-1), Research Centre Jülich, Jülich, Germany; 5 Institut de Biologie Structurale J.-P. Ebel, Université Grenoble Alpes, Grenoble, France; 6 Institut de Biologie Structurale J.-P. Ebel, Centre National de la Recherche Scientifique, Grenoble, France; 7 Institut de Biologie Structurale J.-P. Ebel, Direction des Sciences du Vivant, Commissariat à l'Énergie Atomique, Grenoble, France; University of California, Davis, UNITED STATES

## Abstract

Alzheimer’s disease (AD)-associated amyloid β peptide (Aβ) is one of the main actors in AD pathogenesis. Aβ is characterized by its high tendency to self-associate, leading to the generation of oligomers and amyloid fibrils. The elucidation of pathways and intermediates is crucial for the understanding of protein assembly mechanisms in general and in conjunction with neurodegenerative diseases, e.g., for the identification of new therapeutic targets. Our study focused on Aβ42 and its oligomeric assemblies in the lag phase of amyloid formation, as studied by sedimentation velocity (SV) centrifugation. The assembly state of Aβ during the lag phase, the time required by an Aβ solution to reach the exponential growth phase of aggregation, was characterized by a dominant monomer fraction below 1 S and a population of oligomeric species between 4 and 16 S. From the oligomer population, two major species close to a 12-mer and an 18-mer with a globular shape were identified. The recurrence of these two species at different initial concentrations and experimental conditions as the smallest assemblies present in solution supports the existence of distinct, energetically favored assemblies in solution. The sizes of the two species suggest an Aβ42 aggregation pathway that is based on a basic hexameric building block. The study demonstrates the potential of SV analysis for the evaluation of protein aggregation pathways.

## Introduction

Alzheimer's disease (AD) is an age-related disease with growing incidence in populations with high life expectancies. As a consequence AD poses a major health risk to people and a substantial worldwide economic burden [1]. Therefore, efforts to improve our understanding of the disease mechanism have been taken to develop knowledge-based therapeutic concepts. It is well established that a proteolytic fragment of the amyloid precursor protein (APP) plays a key role in disease pathogenesis, as reviewed in [2,3]. This 39 to 43 residue fragment of the membrane spanning APP, referred to as the amyloid β peptide (Aβ), is highly prone to self-association [4]. Although the monomeric form of Aβ is apparently nontoxic, the self-associated species exhibit neurotoxic behavior [5]. Fibrillar deposits of Aβ in intercellular plaques are a histological marker for post-mortem AD diagnosis and are considered to be the cause of neurodegeneration. More recently, however, it was found that soluble Aβ oligomers are more neurotoxic than the amyloid fibrils and thus may represent the causative agents for neurodegeneration [6–13]. Nevertheless, a conclusive, generally accepted definition of the toxic oligomer is still missing [14]. As pointed out in [15] there are manifold challenges for oligomer study, one of which is the rather low concentrations of the natural oligomeric species in tissues and body fluids of AD patients, which hinder a direct preparation and characterization. Additionally, it is difficult to control Aβ aggregation processes *in vitro*. Therefore structural information that should aid the development of therapeutic interventions is still missing.

Because there is obviously still need for clarification we engaged in this study in a thorough characterization of Aβ aggregation by sedimentation velocity centrifugation (SV), one of the major applications of analytical ultracentrifugation. SV analysis exhibits several advantageous properties, making it especially attractive for the investigation of self-assembling proteins. SV is a first-principles based method. No calibration standards are required as for instance necessary in size exclusion chromatography to assign molecular weights to elution volumes. And even more important the characterization is carried out in solution without a solid phase being involved. In SV analysis all parameters regarding size distribution and shape of aggregates are derived simultaneously from one specific Aβ solution. Although fractionation occurs due to the sedimentation process, leading to improved detectability of the different species, aggregates sediment always in the presence of equilibrium concentrations of smaller species and monomers, preventing dissociation. Sedimentation velocity centrifugation (SV) is suitable for the study of macromolecules ranging from a few thousand Da to several MDa. Measurement of peptide concentrations by absorbance throughout the experiment guarantees control over possible losses of Aβ. In recent years, SV methodology has drastically improved in terms of information content and resolution [16,17]. This is particularly pertinent in the field of aggregating proteins where analytical ultracentrifugation has become an increasingly important technique due to its unique properties [18–25]. In the case of self-assembling proteins, the samples consist of mixtures of differently sized and shaped particles. In SV experiments the solute concentration as a function of the radial position within the rotor is recorded at regular time intervals. These concentration profiles can be fitted by a sum of solutions of the Lamm equation [26]. The *c* ( *s* ) distribution [27] and genetic algorithm based analyses [28] are two implemented software approaches to perform this data fitting task. In previously reported SV experiments, we have demonstrated the power of the data evaluation method for the determination of size- and shape-distributions of the Aβ peptide [29,30].

In our study we decided to investigate the early phase of Aβ42 self-assembly. We chose Aβ42 because the elevation of its levels and of the ratio of Aβ42 to the shorter major form Aβ40, had been identified as important early events in the pathogenesis of AD[31]. Specific questions to be addressed are: what is the size of the smallest detectable oligomer in solution, and do well-defined, oligomeric species exist as the hypothesis of a toxic oligomer might suggest. For this purpose we restricted our analysis to the lag phase of aggregation as defined by thioflavin T kinetic measurements.

## Materials and Methods

### Amyloid β preparation

Synthetic human Aβ42 peptide was purchased as a trifluoroacetate salt (Bachem, Weil am Rhein, Germany). For disintegration of preexisting aggregates, the peptide was predissolved in 100% hexafluoroisopropanol (HFIP) at 1 mg/mL and incubated overnight at room temperature. The solution was then divided into aliquots, lyophilized and stored at -8000B0030C until required. Aβ42 was dissolved at pH 10 for analysis of a monomeric sample. To initiate aggregation the Aβ42 peptide was dissolved at concentrations from 10 to 240 μM in 10 mM NaP_i_ buffer (6.2 mM sodium dihydrogen phosphate, 3.8 mM disodium hydrogen phosphate, pH 7.4).

### Thioflavin T (ThT)-Assay

For the quantitative assessment of amyloid formation, freshly prepared Aβ42 samples in NaP_i_ buffer with 5 μM ThT were incubated in a black 96-well fluorescence plate with an optical bottom (Nunc, Thermo Scientific, Germany). ThT fluorescence was recorded every 30 min in a plate reader (M1000, Tecan, Maennedorf, Switzerland) at *λ*_ex_ = 446 nm and *λ*_em_ = 490 nm with a bandwidth of 5 nm. The temperature was controlled at either 20 or 37°C.

### CD spectroscopy

Circular dichroism spectroscopic measurements were carried out on a Jasco J-815 spectrometer. A 1 mm optical path length cuvette was used. The temperature was controlled at 20°C. For the measurement over time, sample incubation was not performed in the cuvette but in a standard sample tube, from which aliquots were taken. Spectra were recorded from λ = 260 nm to 185 nm at 1 nm resolution, 50 nm/min scan speed, and an integration time of 0.5 s. For signal improvement ten accumulations were averaged. The obtained spectra were transformed from ellipticity θ, measured in millidegrees (mdeg), to mean residue ellipticity (MRE) after subtraction of the buffer spectra.

### Analytical Ultracentrifugation

Sedimentation velocity centrifugation experiments were carried out in a Beckman Optima XL-A (Beckman-Coulter, Brea, CA, USA), equipped with absorption optics and a four-hole rotor. Samples (volume 400 μL) were filled into standard aluminum or epon double sector cells with quartz glass windows. Measurements were performed in the intensity mode [32] at detection wavelengths between 224 and 242 nm in order to adjust for different peptide concentrations. For Aβ42 peptide concentrations above 40 μM the temperature was set to 10°C, otherwise 20°C was chosen. Radial scans were recorded with 20 μm radial resolution at ~1.5 min intervals. The software packages UltraScan II v 9.9/III v 2.0 [28,33] and SEDFIT v 14.1[34] were used for data evaluation. After transformation of the recorded sedimentation velocity data, taken in the intensity mode, to either absorbance or pseudo-absorbance data in the respective data evaluation software, time- as well as radially-invariant noise were calculated and subtracted. In UltraScan a model-independent analysis approach for fitting SV data, which permits simultaneous determination of shape and molecular weight distributions for mono- and polydisperse solutions of macromolecules, was further refined by a parsimoniously regularized fit of independent solutions of the Lamm equation applying a genetic algorithm (GA) to ensure convergence into the global minimum [35]. The final results were subjected to a Monte Carlo (MC) analysis with 50 iterations each. In sedfit, continuous sedimentation coefficient distributions *c* ( *s* ) were determined with 0.05 S resolution and an F-ratio = 0.95. Suitable *s*-value ranges between 0 and 30 S and for GA *f/f*_0_ between 1 and 4 were chosen. Buffer density and viscosity (Table 1) had been calculated with Sednterp v 20111201 beta [36,37]. The partial specific volume of Aβ42 was calculated according to the method of Cohn and Edsall [38,39] as implemented in Sednterp or UltraScan II/III (Table 1). Graphical output for sedfit results was created with the software GUSSI vs 1.0.3, written by Chad A. Brautigam, University of Texas Southwestern Medical Center. All reported *s*-values were corrected for 20°C and water and therefore *s*_20,w_-values.

**Table 1:** Solvent parameters for 10 mM NaPi, pH 7.4 and $\overset{-}{v}$ of Aβ42 for the temperatures used in the SV experiments. Calculations were performed with Sednterp or UltraScan II/III.

temperature (°C)	$\overset{-}{v}$ (cm^3^/g)	density (g/cm^3^)	viscosity (cp)
10	0.734	1.0011	1.3109
20	0.738	0.9996	1.0048

## Results and Discussion

In this study we analyzed freshly prepared solutions of Aβ42 by analytical ultracentrifugation, ThT-assay, and CD spectroscopy to gain insights into the early processes of Aβ42 self-assembly within the lag phase of amyloid formation.

### Monomer characterization at pH 10

**Fig 1:**
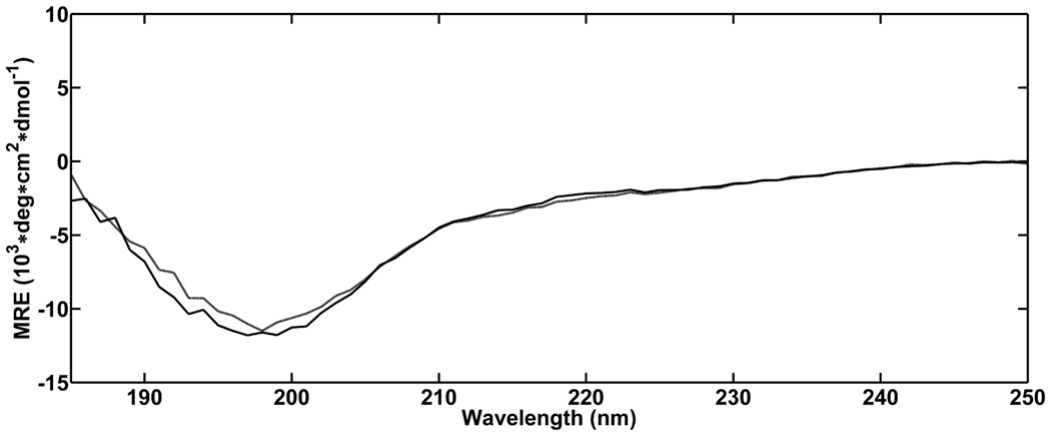
CD-spectroscopy of Aβ42 at pH 10. CD spectra measured at the start of the incubation (black) and after 48 h (grey) at 20°C. Spectra are in agreement with a random coil dominated secondary structure with a characteristic minimum around 200 nm.

In a first step, we wanted to characterize the hydrodynamic properties of monomeric Aβ42. At basic pH Aβ42 is stable as a monomer [40]. In contrast to organic solvents like HFIP or TFE [41] known for their monomer stabilizing properties the chosen basic conditions do not induce an α-helical structure. At pH 10 (Fig 1) the Aβ42 peptide shows a secondary structure profile comparable to the one determined at pH 7.4 for the earliest time point as shown in Fig 1. The CD spectrum of the peptide at basic pH did not change within 2 d of incubation, indicating the required stabilization of the peptide. To increase the certainty of species detection, we compared two different software packages for analytical ultracentrifugation data evaluation, i.e., sedfit [34] and UltraScan [33].

**Fig 2:**
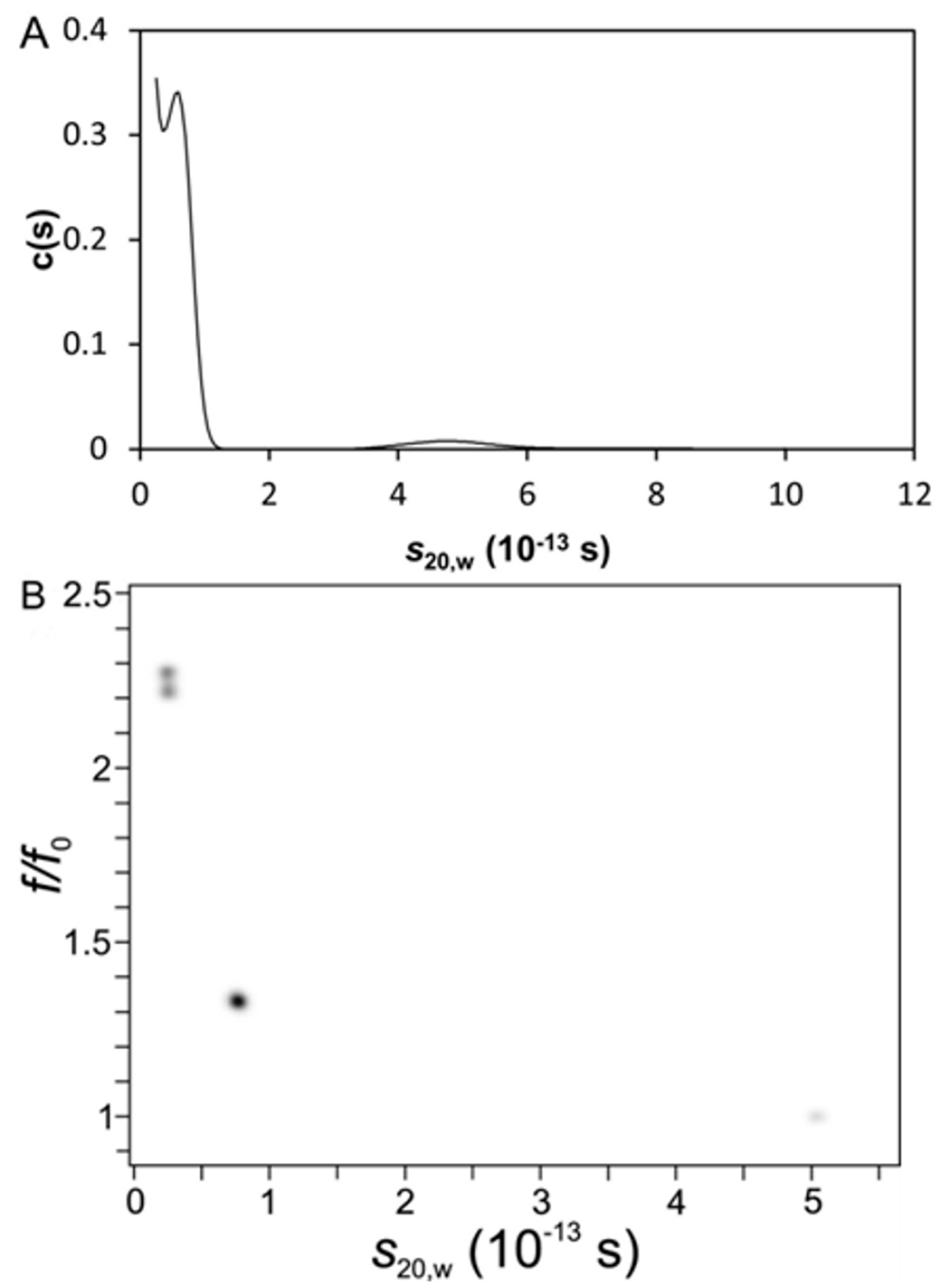
SV-analysis of Aβ42 at pH 10. 40 μM Aβ42 had been centrifuged at 40,000 rpm and 20°C. By *c*(*s*) analysis the most prominent species was detected at s = 0.6 S (A); GA-MC analysis revealed the dominant species at *s* = 0.67S with *f/f*_*0*_ = 1.35 (B). Results are presented in a pseudo-3D plot with a color coded third dimension indicating the species fraction. In both evaluations a small amount of near spherical aggregates appeared at 5 S. The minor species detected below 1 S are artifacts due to a base line deconvolution problem.

**Fig 3:**
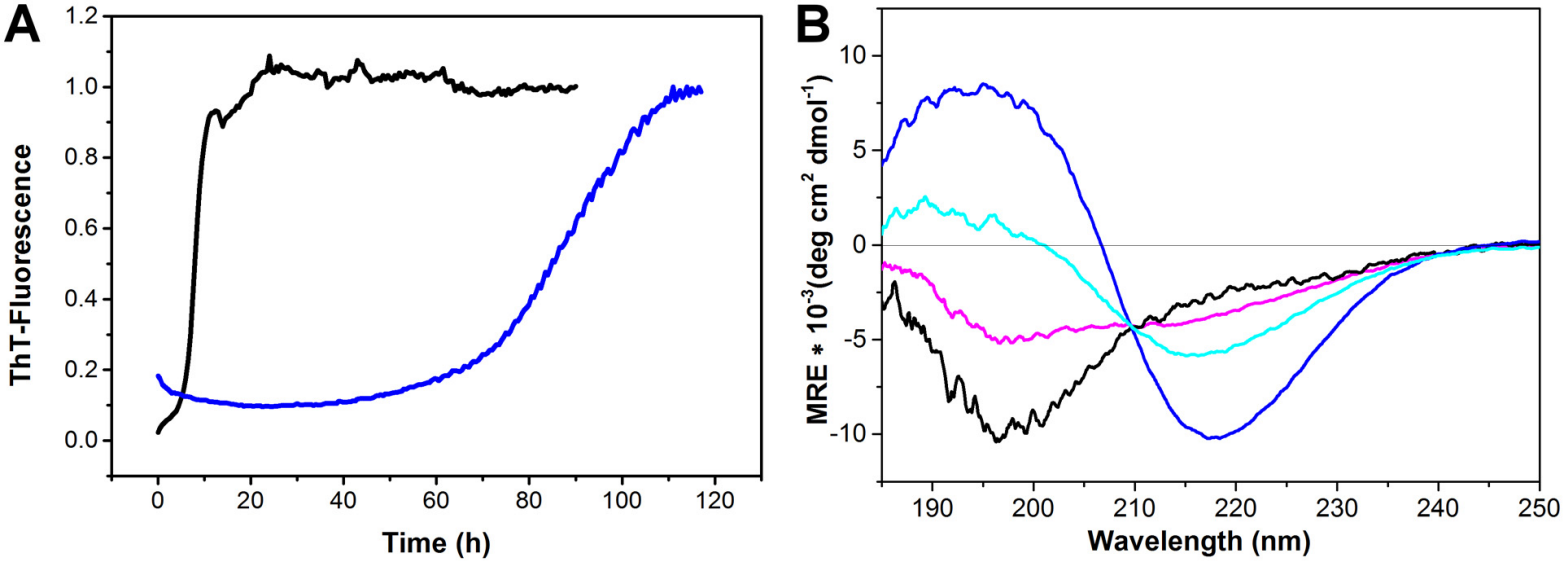
Kinetics of amyloid formation. ThT-fluorescence kinetics of 40 μM Aβ42 in 10 mM NaP_i_ at 37°C (black) and 20°C (blue). The maximum RFUs had been normalized to 1. Determined lag times were ~8 h at 37°C and ~80 h at 20°C (A). CD spectra of 37 μM Aβ42 incubated at 20°C for 0 (black), 29 (magenta), 49 (cyan) and 74 h (blue). The spectrum of Aβ42 in NaP_i_, pH 7.4 changes within the indicated times from a predominantly random coil spectrum to a β-sheet rich spectrum. Superimposed spectra possess an isosbestic point at 209 nm, indicating a two-state transition (B).

The SV analysis of 20 μM Aβ42 at pH 10 revealed that the Aβ42 solution consisted of 95% monomers. In Fig 2 the calculated *c* ( *s* ) distribution shows a first peak at 0.6 ± 0.2 S, representing 94% of the sample, and a second broader peak at 4.8 ± 0.5 S, accounting for 6%. The signal increase at *s*-values between 0 and 0.6 S indicated either the presence of smaller species than Aβ42, i.e., fragments that we could neither identify in gel electrophoresis nor mass spectrometry or, more likely, a baseline deconvolution problem caused by the small size of the macromolecules, preventing clearance of the meniscus region during centrifugation. The 0.6 S species fits well to monomeric Aβ42, the 4.8 S species indicates an oligomer of 50 to 70 kDa. The weight averaged *f/f*_0_ was fitted as 1.56. According Eq. 1 the 0.6 S species with *f/f*_0_ = 1.56 had a molecular weight of 4.4 kDa, which is close to the molecular weight of the monomeric Aβ42 (4.5 kDa). In contrast to the *c* ( *s* ) determined *s*-value distribution (Fig 2) the GA-MC analysis in UltraScan (Fig 3) fitted three species with an *s*-value smaller than 1, which differed significantly with respect to their frictional ratios. The main species with 0.67 S had a smaller *f/f*_0_ value of 1.35 than the main component from the *c* ( *s* ) analysis. Again the resulting molecular weight is close to 4.6 kDa, which confirmed the monomeric nature of the species. Taken together around 6% of the aggregates with an average *s*-value of 5 S were detected independent from the data evaluation procedure. These aggregates might comprise either residually undissolved material or newly formed assemblies.

The experimentally determined *s*-value of the monomer is in agreement with an *s*-value of 0.55 S reported in the literature [42]. In the PDB a high resolution structure of full-length Aβ42 exists, that was determined by NMR in an HFIP-water mixture (1Z0Q) [43]. The *s*-value calculated for a bead model [44,45] built from this NMR structure of Aβ42, is 0.72 S and the frictional ratio is 1.3. The structure is characterized by 42% helices and several turns giving it a more compact conformation. In comparison we can conclude that the Aβ42 monomer at pH 10 characterized in our experiments had a more elongated shape, i.e., it was less structured than the structure deposited in the PDB. This result is in agreement with the peptide adopting a random-coil dominated structure, when the helix inducing agent HFIP is not present in the sample buffer.

### Length of the lag phase

An SV experiment for Aβ42 monomers at 20°C and 60,000 rpm corresponding to 257,000 g takes 6 to 10 h, because of the slow sedimentation of the monomer. If assemblies present in the lag phase are to be characterized, the lag phase has to last longer than the experimental analysis. Entry into the rapid growth phase would also cause profound changes to the sample composition, thereby complicating data analysis. To determine the length of the lag phase, we followed the process of amyloid formation starting from freshly dissolved Aβ42 by a ThT fluorescence assay (Fig 3A). Measurement over time typically results in a sigmoidal curve, that is subdivided into the lag phase during the initial stage, a steep increase in fluorescence during the growth phase, that represents the formation of amyloid fibrils, and finally the plateau region, where fluorescence is constant and then decreases slightly after a period due to precipitation. The duration of the lag phase was about 80 h for 40 μM Aβ42 (Fig 3A) at 20°C. To complement the fluorescence data, the structural conversion from random coil to β-sheet structure, which accompanies self-assembly of monomeric Aβ42 units to fibrils with an amyloid specific cross-β conformation, was monitored by CD spectroscopy. In Fig 3B, CD spectroscopic measurements over time at 20°C for a 37 μM Aβ42 solution indicated a conformational change, characterized by the presence of an isosbestic point, within 49 to 74 h. The isosbestic point at 209 nm suggests a two-state transition between random coil and β-sheet conformation. The duration of the lag phase determined by the CD measurements and the ThT assay were within the same order of magnitude. Shortening of the lag phase with increasing initial Aβ42 concentrations was compensated by reducing the temperature from 20 to 10°C during SV analysis of samples at concentrations higher than 40 μM. It was therefore possible to perform the SV experiments within the lag phase of the system and before the initiation of the rapid growth phase.

### Concentration dependent self-association

**Fig 4:**
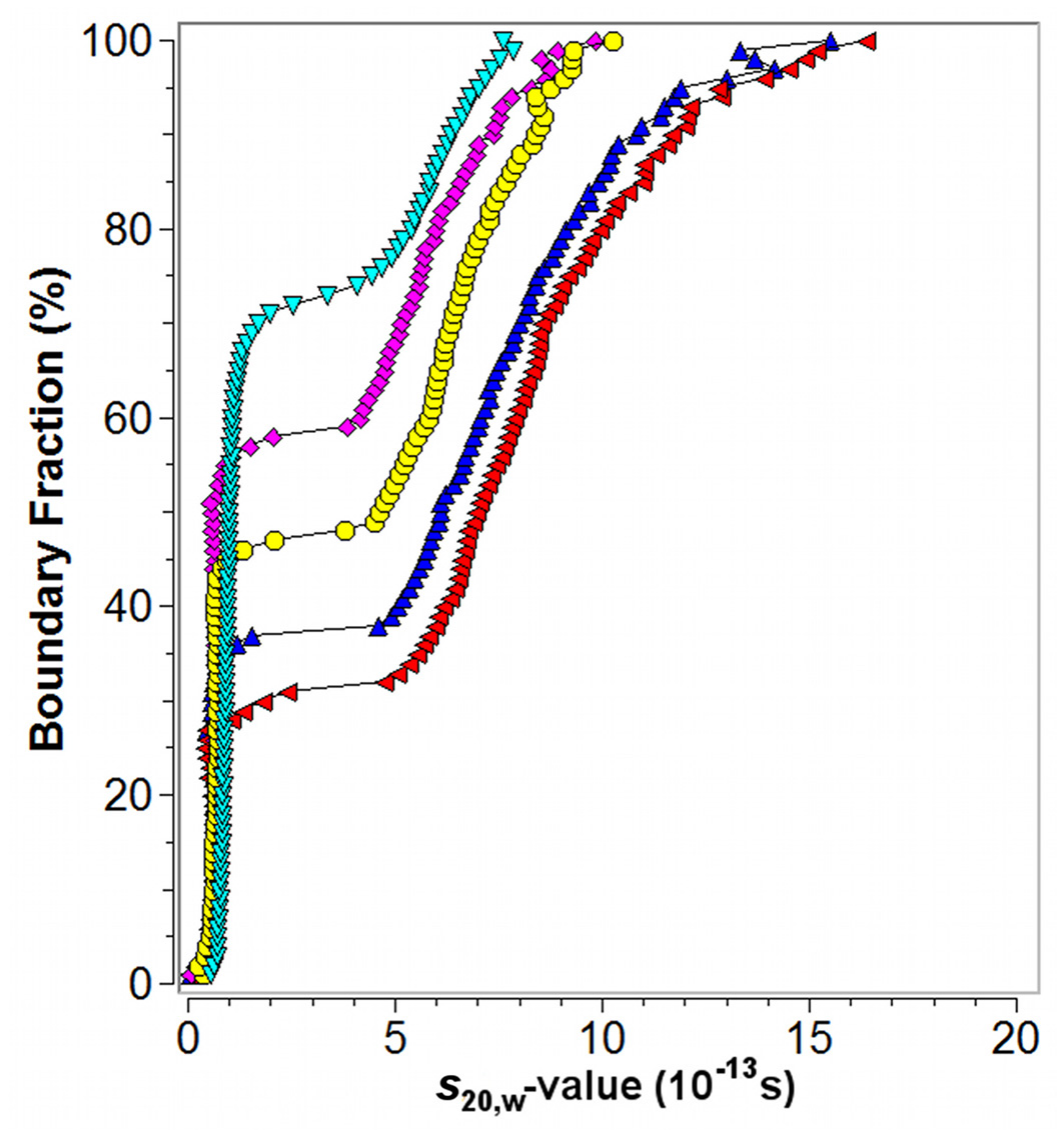
G(s) distributions for different initial concentrations of Aβ42. Model independent analysis of SV data by the van Holde-Weischet method for freshly prepared Aβ42 solutions with 40 μM (cyan triangle), 80 μM (magenta diamond), 160 μM (yellow circle), 200 μM (blue triangle upwards), and 280 μM (red triangle leftwards) initial monomer concentrations.

Samples were prepared by freshly dissolving HFIP pretreated synthetic Aβ42 at different initial concentrations. At the start of the sedimentation process all samples had a history of ~1 h handling and thermal equilibration time. Calculation of the initial peptide concentration from a first scan at 3000 rpm and the first scan at 55,000 and 60,000 rpm, respectively, revealed a deficit between 20 and 25% for all samples. Material might have been lost to inter-/surfaces, undissolved, and/or sedimented during the acceleration phase. In a first approach, the SV data were evaluated in a model independent manner by the van Holde-Weischet (vHW) method [46]. Fig 4 shows the combined vHW-distribution plots for 40, 80, 160, 200 and 280 μM initial Aβ42 concentrations. Analyzed solutions showed bimodal distributions characterized by a slowly sedimenting, rather homogenous fraction, visible at the near vertical line in the vHW-distribution at small *s*-values, and a clearly separated, faster sedimenting fraction with *s*-values ranging between 4 and 15 S. The Aβ42 species detected at *s*-values below 1 S were assigned to monomeric species based on the monomer characterization at pH 10 (Fig 2). The proportion of oligomeric species was found to increase from ~30% to ~50% without populating oligomeric species larger than 20 S by a sevenfold increase of the initial Aβ42 concentration of 40 μM. Under the applied experimental conditions, that is speed, temperature, and solvent, molecular species up to 200 S would be detectable in SV analysis. As a consequence the weight averaged *s*-value of the oligomer fraction increased with increasing Aβ42 concentration. The near horizontal curve progression between *s*-values 1 and 4 S indicates the absence of detectable Aβ42 assemblies in this area and marks therefore the range where kinetically unstable intermediates might be located. Sedimenting species with *s*-values between 1 and 4 S would correspond to oligomers ranging from dimer to octamer assuming a spherical shape, thus giving lower size limits of the intermediates. A further observation was a slight shift of the monomer peak to smaller *s*-values for those measurements performed at 10°C when compared with the data recorded for a 40 μM Aβ42 sample at 20°C. The observed change in *s*-value could be the result of a temperature induced conformational change leading to an altered frictional coefficient of the monomer in solution. Alternatively or additionally the shift might be due to the presence of an unresolved fast equilibrium, e.g. between the monomer and a dimer.

**Fig 5:**
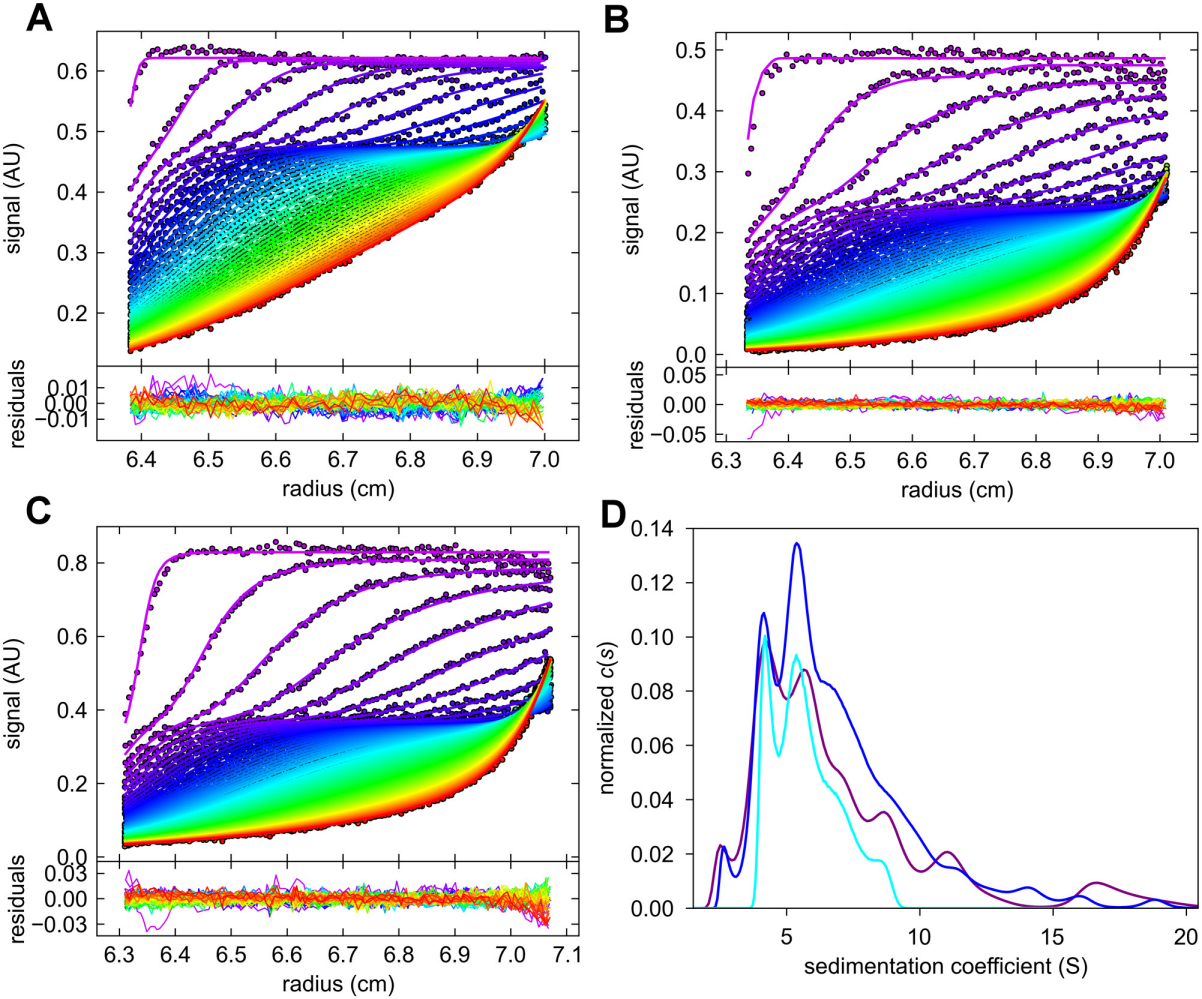
SV analysis of Aβ42 at different initial concentrations. The noise corrected data (dots) superimposed with fitted curves (lines) from *c* ( *s* ) analysis, and the residuals plot attached below, are shown for 40 μM (A), 80 μM (B), and 160 μM (C) initial concentrations of Aβ42. For clarity, only every second scan and every third data point is shown. The combined *c* ( *s* ) distributions for 40 μM (cyan) measured at 20°C, 80 μM (magenta) measured at 10°C, and 160 μM (blue) measured at 10°C are shown in (D). The monomer peak below 1 S has been excluded from the plot.

Our major objective was to identify discrete Aβ42 oligomeric species. Such species should feature energetically favored assemblies consisting of a well-defined number of monomeric units. To identify single species, the data were evaluated by calculating *c* ( *s* ) distributions, as well as by applying a parsimoniously regulated genetic algorithm, as implemented in UltraScan. For both approaches the underlying models assume the existence of a number of independent species in solution. For a rapid equilibrium with the smaller compound being present in excess as is the case in our system *c*(*s*) is a valid approximation for data evaluation [47]. Provided that seed formation in the lag phase is a slow and rare process, the solution state could be approximated as steady-state equilibrium. This assumption was corroborated by small root mean square deviation (rmsd) values obtained by measuring the agreement between measured and calculated data, which amounted to ~0.5% of the total signal. In Fig 5 the noise subtracted raw data together with the fitted curves of SV runs with three different initial Aβ42 concentrations (A, B and C) and the combination of the resulting *c* ( *s* ) distributions (D) are shown.

**Fig 6:**
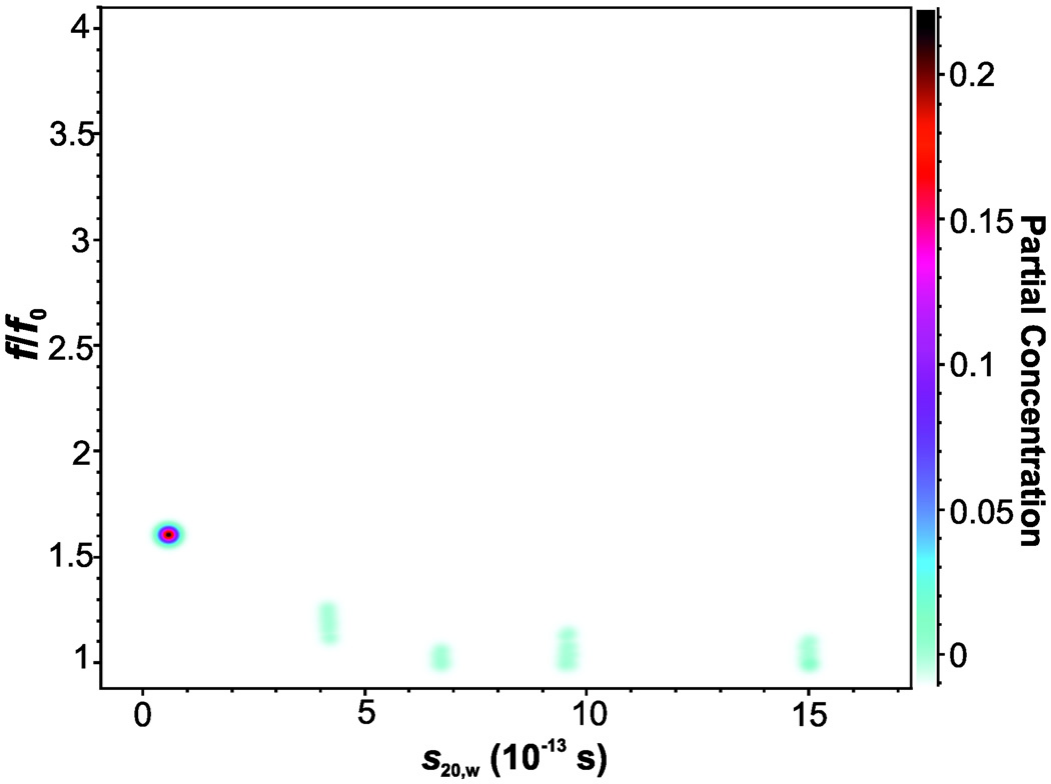
Pseudo-3D plot of Aβ42 size and shape distribution. Results from a SV analysis of 280 μM Aβ42 at 60,000 rpm and 10°C. Data were evaluated by 2-dimensional spectrum analysis followed by GA-MC analysis.

As part of the oligomer fraction two prominent species were repeatedly detected by *c*(*s*) analysis (Fig 5D) with *s*-values averaged for 9 independent samples of 4.70 S ± 0.27 S and 6.25 S ± 0.28 S (Table 2). These two species were observed under different experimental conditions, i.e., initial Aβ42 concentration, rotor speed and temperature. A third species appeared as a shoulder of the larger species with an *s*-value of 6.2 to 7.6 S. The smaller species at 2.6 S, and any species larger than 10 S were detected at too low concentrations to be reliably assigned. In *c* ( *s* ) a global *f/f*_*0*_ weight averaged for all *s*-value species was determined. As already noticeable in the vHW-distribution (Fig 4) the percentage of oligomeric species in the range between 1 and 15 S increased with increasing initial Aβ42 concentrations, while concomitantly weight averaged *f/f*_*0*_ decreased from 1.6 to 1.2 (data not shown). This indicates an increase of globular particles present in solution. To resolve individual shape parameters data evaluation was performed by applying GA in UltraScan. The monomeric species showed a slightly extended conformation with an *f*/*f*_0_ of 1.6, in agreement with the results from SV analysis at pH 10 (Fig 2). For the oligomers ranging from 4 to 10 S, *f*/*f*_0_ values < 1.3 were calculated, which is consistent with the results from *c*(*s*) showing less extended conformations with increasing fractions of oligomeric species. Although the determined *s*-values for the two species could be reproduced by the GA algorithm (Fig 6), the Monte Carlo statistics for the GA results revealed considerable uncertainties for *f*/*f*_0_ determinations, probably due to the comparatively low number of scans reporting about the sedimentation and diffusion properties of the larger species. Although no conclusive *f*/*f*_0_ values for single species could be retrieved, both methods support a globular shape of the oligomers. The obtained shape information had been confirmed by AFM measurements (data not shown). Additionally it is in agreement with reported AFM and TEM studies from literature [48,49].

**Table 2:** Observed *s*-value species of Aβ42. The oligomeric state was determined for a globular particle with *f*/*f*_0_ = 1.1. The error was calculated as standard deviation of the mean of *s*-values obtained by applying the c(s) routine to the given number of different data sets. Different data sets correspond to independent sample preparations.

	*s*-value [10^–13^ s] ± SD	Number of data sets
Monomer	0.62 ± 0.02	12
12-mer	4.70 ± 0.27	9
18-mer	6.25 ± 0.28	9

Calculations of the molecular weights of the oligomers based on the determined *s*-values utilizing shape factors (*f/f*_0_) between 1.0 and 1.1, typical for globular proteins, using the following equation

$$s_{20,w}^{0}\;=\;\frac{\left(1-\overset{-}{v}\rho \right)}{\left(f/f_{0}∙6\eta \right)}∙\sqrt[{3}]{\frac{4}{3\overset{-}{v}}{\left(\frac{M∙n}{\pi ∙N_{A}}\right)}^{2}}$$

with solvent density (*ρ*) and viscosity (*η*) revealed for the 4.7 S species a size corresponding to 11 to 13 Aβ42 monomeric units (*n M*, *M* molecular weight of Aβ42) and for the 6.25 S species accordingly 17 to 20 monomeric Aβ42 units. The third species in the range of 6.2 to 7.6 S covers oligomeric species built from 22 to 29 monomeric units.

The determined sizes further supports prior evidence of a hexameric building block in Aβ42 aggregation [50–52] A hypothetical Aβ42 hexamer with an *s*-value between 1.9 (*f*/*f*_0_ = 1.6) and 3.1 S (*f*/*f*_0_ = 1.0) would be located within the predetermined gap between 1 and 4 S. The hexamer is not detected in solution because it is a kinetically unstable intermediate. The fraction of oligomeric species in solution is concentration dependent and reached 75% at 280 μM Aβ42 initial concentration; taking the 25% loss of Aβ42 during acceleration into account the fraction is 56%.

Although the described oligomers were detected free in solution for the first time, their sizes agree well with previously reported species. A dodecamer was detected by PICUP [6,52] and confirmed by ion mobility spectrometry-mass spectrometry [53]. Dodecameric assemblies were also described as Aβ-derived diffusible ligands and a specific assembly detected in cerebrospinal fluid, Aβ*56. Additionally, the oligomeric species termed globulomer [54] is a dodecamer, although the preparation of this species required SDS for maintaining stabilization. Experimental and theoretical evidence from MD simulations exists for the 18mer [55–57]. In contrast to our work, the 18mer reported in these references is dependent on hydrophobic interactions either with lipids in a bilayer or fatty acids. Kumar *et al*. [56] showed *c* ( *s* ) distributions for their large fatty acid-derived Aβ oligomers with peaks at 5 and 7 S, which are similar to our *c* ( *s* ) distributions obtained for Aβ42.

In our study of Aβ42, oligomerization clearly preceded the conversion to a β-sheet conformation. This is in contrast to an analytical ultracentrifugation based study of the prion protein (PrP), where the detected small oligomers showed significantly larger shape factors *f*/*f*_0_ than the PrP monomer, and oligomerization happened in the same time frame as the structural conversion to a β-sheet dominated structure as demonstrated by CD measurements [24]. The 12-mer and the 18-mer were detected reproducibly under different speed conditions, temperature and initial Aβ42 concentrations. With increasing initial Aβ42 concentrations the fraction of these oligomers increased rather than larger aggregates forming. This is the first time that these species were detected free in solution. We suggest that these species are part of an oligomer formation pathway similar to the one proposed by Barghorn *et al*. [54] based on hexameric building blocks; although we believe that the two pathways for either fibril or oligomer formation are not completely separated from each other. To resolve this question, the addition of oligomers to monomeric Aβ solutions must be tested to determine whether a decrease in the lag phase is observed. Additionally, it should be clarified whether the observed oligomers are cytotoxic towards neuronal cells. Therefore, oligomers have to be purified away from monomers and larger assemblies.

## Conclusions

The pathogenic aggregation mechanism of the amyloid beta peptide is still a matter of debate. Especially the nature of a postulated oligomer responsible for the neurodegeneration in Alzheimer's disease is elusive. In this study we utilized a technique that allows for observation of all species present within a sample at a given time point without complications arising from involvement of solid phases or necessity for any calibration. Size, shape, and quantity of all observed *s*-value species could be determined simultaneously, avoiding unaccounted changes in sample composition in the course of multi-step analysis procedures. In contrast to our expectations it was possible to increase the initial Aβ42 concentration above 1 mg/ml without generating larger aggregates and fibrils within the experimental time frame. This was caused or at least supported by the reduced temperature of 10°C and the property of the SV method to deplete solutions of larger aggregates, which might act as seeds for amyloid formation. Such particles with high *s*-values are removed by their rapid sedimentation during the early phases of centrifugation, i.e., by their rapid sedimentation already during the acceleration phase. We were able to demonstrate the existence of distinct oligomeric species in solutions of Aβ42 during the lag phase of amyloid formation. These oligomers appeared to be globular in shape, while the monomer showed a slightly extended conformation. During the experiment, starting from a homogeneously filled cell, the characterized oligomers sedimented always in the presence of smaller species, thus maintaining equilibrium between distinct oligomeric species and smaller oligomers and monomers. The postulated equilibrium is in agreement with the observation that increasing the initial Aβ42 concentration led rather to an increase of the oligomer fraction than to the appearance of aggregates above 20 S.

The two recurring species were interpreted as an 11 to 13mer for the smaller and a 17 to 20mer for the larger oligomer assuming a frictional ratio between 1.0 and 1.1. The numbers are tempting to suggest a model based on a hexameric building block as recently proposed in [58]. The high reproducibility of the *s*-values as well as their independence from the initial Aβ42 concentration clearly indicates the existence of energetically favored assemblies among possible oligomeric states. It can be assumed that the defined size of the oligomers correlates with an equally well defined secondary and tertiary structure. Such characterized species would be ideal candidates for specific, pathogenic interactions with cellular receptors or other interaction partners. Whether the observed oligomers are on or off-pathway to the amyloid fibrils cannot be concluded, although it seems probable that once a certain number of monomeric units are assembled this might be the place where—triggered by a conformational rearrangement—the rapid growth of fibrillar structures initiates.
